# Quantitative ELISA-Like Immunohistochemistry of Fibroblast Growth Factor 23 in Diagnosis of Tumor-Induced Osteomalacia and Clinical Characteristics of the Disease

**DOI:** 10.1155/2016/3176978

**Published:** 2016-03-13

**Authors:** Fangke Hu, Chengying Jiang, Qiang Zhang, Huaiyin Shi, Lixin Wei, Yan Wang

**Affiliations:** ^1^Department of Orthopedics, Tianjin Hospital, Tianjin 300211, China; ^2^Department of Breast Cancer Pathology and Research Laboratory, Tianjin Medical University Cancer Institute and Hospital, Tianjin 300060, China; ^3^Department of Orthopedics, Chinese PLA General Hospital, Beijing 100853, China; ^4^Department of Pathology, Chinese PLA General Hospital, Beijing 100853, China

## Abstract

Tumor-induced osteomalacia (TIO) is a rare acquired paraneoplastic disorder and fibroblast growth factor 23 (FGF23) plays a key role in its pathogenesis. This study was conducted to describe a novel FGF23 detecting procedure and describe clinical features of the disease. Fourteen TIO cases were retrieved and FGF23 expression was measured by quantitative ELISA-like immunohistochemistry using formalin-fixed and paraffin-embedded tissues. As summarized from 14 TIO cases, clinical features of TIO were long-standing history of osteomalacia, hypophosphatemia, and urinary phosphate wasting. The associated tumors were mostly benign phosphaturic mesenchymal tumors mixed connective tissue variant (PMTMCT) which could be located anywhere on the body, and most of them could be localized by conventional examinations and octreotide scanning. By quantitative ELISA-like immunohistochemistry, all the 14 TIO cases had high FGF23 expression (median 0.69, 25%–75% interquartile 0.57–1.10, compared with 26 non-TIO tumors of median 0.07, 25%–75% interquartile 0.05–0.11, *p* < 0.001). The quantitative ELISA-like immunohistochemistry was a feasible and reproducible procedure to detect the high FGF23 expression in formalin-fixed and paraffin-embedded biopsies or specimens. Since TIO was often delay-diagnosed or misdiagnosed, clinicians and pathologists should be aware of TIO and PMTMCT, respectively.

## 1. Introduction

Tumor-induced osteomalacia (TIO) or oncogenic osteomalacia (OOM) is reported to be a rare acquired paraneoplastic disorder caused by renal phosphate wasting and characterized by hypophosphatemia and osteomalacia [1–5]. Since McCance [6] described the first case in 1947, more than 300 cases could have been retrieved in the literature [7]. As the majority of the disease has not been diagnosed or reported, it is highly probable that the prevalence of TIO is underestimated [3].

The causative tumors of TIO are mostly located in bones, soft tissues, and craniofacial sinuses [2]. Pathologically most of them are phosphaturic mesenchymal tumors mixed connective tissue variant (PMTMCT) which are usually misdiagnosed as other tumors, such as hemangiopericytomas, giant cell tumors, osteoblastomas, and chondroblastomas [4]. As PMTMCTs are mostly benign tumors, normalization of serum phosphate and remission of osteomalacia could be achieved if the causative tumors are completely removed [4, 8, 9]. The definite diagnosis and location of the responsible tumor appears especially essential [3].

By inhibition of renal tubular epithelial phosphate transport, phosphaturic hormone of fibroblast growth factor 23 (FGF23) plays a key role in the pathogenesis of TIO [2, 4, 5]. Serum FGF23 concentrations are significantly elevated in TIO patients while decreasing dramatically after complete tumor removal (the reported plasma half-life was 46–58 min [10]). Besides the increased serum FGF23 level, high FGF23 mRNA transcription and protein expression could also be detected in tumor specimens [2, 11, 12]. As the majority of specimens for clinical diagnoses and researches are stored as formalin-fixed and paraffin-embedded blocks, the detection of the high FGF23 expression in these tissues is of great significance. While commercial antibody of FGF23 for immunohistochemistry staining does not work well and mRNA extracted from specimen is easily degradated, we documented a novel FGF23 detecting procedure using paraffin-embedded tissues in the present study. We compared the result with the previously reported FGF23 Reverse Transcription-Polymerase Chan Reaction (RT-PCR) assay [2] and also detailed the clinical, laboratory, and pathological characteristics of the included TIO cases.

## 2. Methodology

### 2.1. Patients and Analysis Strategy

TIO cases were retrieved from the Clinical Database of the Chinese PLA General Hospital from 2003 to 2012. Either diagnosed TIO/PMTMCT cases or cases with the following characteristics were included: (1) clinical symptoms of osteomalacia such as proximal muscle weakness and bone pain, (2) absence of personal and familial history of hypophosphatemic disorders, and (3) typical preoperative biochemical parameters such as hypophosphatemia and renal phosphate wasting which returned normal after complete tumor resection [9, 13, 14]. Patients with no surgical specimen available or patients unwilling to be involved in the study were excluded. A control group of tumor specimens where PMTMCTs were usually misdiagnosed was also selected.

We collected the demographic data, clinical features, biochemical results, intraoperative findings, and postoperative outcomes of the included cases. Maximum tubular reabsorption of phosphorus factored for glomerular filtration rate was calculated according to the reported method [15]. (TmP/GFR represents whether phosphate is excreted or absorbed. When serum phosphorus is low, TmP/GFR should be relatively high in order to reabsorb more phosphate in a compensatory mechanism.) Histopathologic specimens were reviewed by two pathologists (Chengying Jiang and Huaiyin Shi) without knowledge of the original diagnoses and further diagnoses were made when consensus was reached. Followup was done on telephone by an orthopedist (Fangke Hu). The study was approved by local Institutional Review Boards and informed consents were obtained from all the patients or their relatives. Data were analyzed by SPSS software (version 16.0; SPSS, Inc., Chicago, Illinois). Data distribution was tested by Kolmogorov-Smirnov Test. For variables of normal distribution, means ± standard deviations were given, while others were shown by medians with 25%–75% interquartiles. The comparison of the absorbance of Quantitative ELISA-like immunohistochemistry was tested by Mann-Whitney test and two-sided *p* < 0.05 were considered of statistical significance.

### 2.2. Quantitative ELISA-Like Immunohistochemistry

Quantitative ELISA-like immunohistochemistry was performed on formalin-fixed and paraffin-embedded tissues. Sections (4 *μ*m in thickness) were trimmed into a core (1 cm in diameter) on slides by scalpel and then deparaffinized and rehydrated. Epitope retrieval was performed in citrate buffer (pH 6.0) at 120°C for 2.5 min. Then the slides were exposed to 3% hydrogen peroxide for 20 min and washed by PBS buffer 5 min for 3 times. A circle was drawn with an ImmEdge hydrophobic barrier pen on the slides around the tissues and further blocking was performed with normal goat serum for 30 min. The component of horseradish peroxidase (HRP) conjugated FGF23 antibody (Cat. # 40-6120) of the Human FGF23 ELISA kit (Immutopics, Cat. # 60-6100) was used. The purified goat polyclonal FGF23 antibody was conjugated to enzyme HRP and could detect epitopes within carboxyl-terminal portion of FGF23. After incubation of the HRP conjugated FGF23 antibody with the sample, slides were washed and incubated with ELISA HRP substrate and then measured in a spectrophotometric microtiter plate reader. The detailed procedure was as follows: 20 *μ*L HRP conjugated FGF23 antibody diluted by 10 *μ*L PBS was added to the sample, sealed, and incubated at room temperature for 4 hours on a horizontal rotator. Then the slides were washed by PBS for 5 min and repeated 5 times. The ImmEdge hydrophobic barrier pen circle was drawn again and 90 *μ*L ELISA HRP substrate (tetramethylbenzidine with hydrogen peroxide) was added and resealed and incubated at room temperature for 30 min on a horizontal rotator. Then transfer 30 *μ*L of the reaction solution to a 96 well plate, and read the absorbance at 620 nm against a blank HRP substrate taken as wavelength correction. Stop the reaction with 0.36 M sulfuric acid and read the absorbance at 450 nm against a reagent blank. Each specimen was measured thrice at different times.

### 2.3. RT-PCR

The FGF23 mRNA transcription was detected by RT-PCR assay both in TIO specimens and the control tumors. Total RNA was extracted from paraffin-embedded sections with TRIzol Reagent kit (Cat. 15596-018, Invitrogen) following a previous reported procedure [16]. For RT reaction, a FGF23-specific primer was designed which spanned the intron/exon boundaries: 5′-CTCTGGGTCTGTGCCTTGT-3′ (forward) and 5′-TGTTGCCTCTGAAATCCATG-3′ (reverse). The 313 bp RT product spanned all the 3 exons of FGF23 gene (Genbank ID: NM_0220638). The specified product was reverse-transcribed from 50 ng of total RNA in a volume of 20 *μ*L, using the ProtoScript M-MuLV Taq RT-PCR Kit (#E6400S, New England Biolabs) according to manufacturer's instructions. The housekeeping gene of phosphoglycerokinase (189 bp) was coamplified to check the RNA quality [2]. PCR procedure was detailed elsewhere [2] and the above specified primers were applied. The PCR products were fractionated by 2% agarose gel electrophoresis and sequenced.

## 3. Results

### 3.1. Patients Included

Between 2003 and 2010, there were about 400,000 hospitalized patients at our institution. From the clinical database, we retrieved 5 cases of diagnosed TIO or phosphaturic mesenchymal tumor (PMT). Other 11 cases with typical clinical and laboratory characteristics were also retrieved, two of which were excluded for no surgical specimen available. Eventually there were 14 cases included, 6 males and 8 females, with a mean age of 39.0 ± 10.8 years (18–55 years) at the time of surgery (case  11 was previously reported elsewhere [17]).

### 3.2. Clinical Findings

Detailed in Table 1, a long-standing history of osteomalacia was predominant in most of the cases except case  13. The duration of osteomalacia (from the onset of symptoms to surgery) was 3.9 ± 2.5 years (1.5–10.0 years). The initial clinical symptom was usually nonspecific bone pain, including low back pain (8 cases), knee pain (3 cases), hip pain (2 cases), ankle pain (2 cases), and costal part pain (2 cases). Because of the renal phosphate wasting and disorder of calcium metabolism, osteoporosis would progress gradually over the years, and eventually patients were bedridden because of bone pain of the whole body and weakness of muscles. Bone pain of the whole body, muscle weakness, and walking difficulty were presented in most cases (12/14, 9/14, and 12/14 resp.), and the process from the onset of pain to these manifestations involved with medians of 1.3 years (1.0–3.0 years), 1.0 year (0.5–4.2 years), and 1.0 year (0.5–3.0 years), respectively. Osteoporotic fractures were common (9/14 cases and 5 of which were multiple fractures), including vertebral compression fractures (5 cases), multiple rib fractures (4 cases), and femoral neck fractures (2 cases). There were 3 cases with bilateral femoral heads necrosis. Most cases had initial hospitalization (median 2.0 times) before final diagnoses. Eventually 6 tumors were localized by palpation, each case by ultrasound, X-ray, CT, and MRI. The other 4 tumors which couldn't be identified by the conventional examinations were localized by octreotide scanning. Octreotide scanning was positive in 11/11 TIO tumors available.

**Figure 1:**
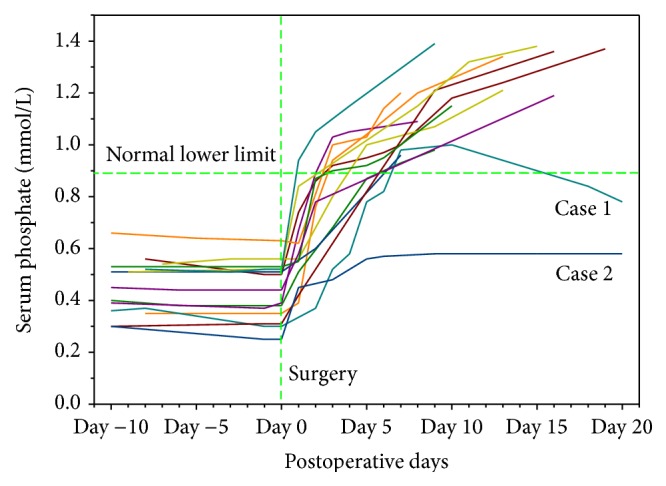
Preoperative and postoperative serum phosphate levels of 14 included TIO cases.

Six of the 14 TIO tumors were localized in bones, 6/14 in soft tissues and 2/14 in craniofacial sinuses. They were measured at 1.0–14.0 cm in the maximum dimension (median 3.0 cm, 25%–75% interquartile 2.0 cm–3.3 cm). During the postoperative follow-up periods of 0.7–8.0 years (median 2.1 years, 25%–75% interquartile 1.7 years–3.5 years), renal phosphate wasting and clinical symptoms of osteomalacia for 11/14 cases had resolved. Bone pain had disappeared at 1.0–2.0 months (median 1.5 months) and they had walked again at 0.5–3.5 months (median 2.0 months). No recurrence occurred for the 11/14 cases and their chemical examinations were normal. As shown in Figure 1, case  1 underwent a surgery of curettage and bone graft. Due to surgery residue, local recurrence occurred at 1.5 months and total hip arthroplasty was performed. In case  2, the carotid artery and cavernous sinus were inflicted, partial resection was performed, and unresectable recurrence occurred at 3 months and no further surgery could be performed. Then octreotide therapy was tried; however, no effect could be detected. Case  4 developed into local recurrence at 2.5 years and the tumor was further resected.

### 3.3. Biochemical Findings

As detailed in Table 2, TIO cases were characterized by low serum phosphate level (mean 0.52 ± 0.09 mmol/L), high serum alkaline phosphatase level (ALP, mean 265.5 ± 172.0 U/L), normal or mild low serum calcium level (normal in 8/14 cases and low in 6/14 cases, mean 2.25 ± 0.09 mmol/L), normal or high parathyroid hormone level (PTH, normal in 7/13 cases and high in 6/13 cases, mean 7.98 ± 5.90 pmol/L), high urine phosphate level (mean 19.1 ± 8.4 mmol/24 h), low or normal urine calcium level (low in 10/13 cases and normal in 3/13 cases, mean 1.89 ± 1.71 mmol/L), and low TmP/GFR (mean 0.46 ± 0.10 mmol/L). As presented in Figure 1, postoperative serum phosphate level increased dramatically. The mean period of the serum phosphate returning to normal level was 3.5 ± 1.8 days (0.9–6.0 days). Case  1 and case  2 whose tumors were only partly resected presented with transitory normal or still low postoperative serum phosphate levels.

### 3.4. Histopathological Findings

As presented in Table 1, the 14 included tumors were initially diagnosed as 5 PMTs, 3 giant cell tumors of tendon sheath, 1 angiofibroma, 1 hemangioendothelioma, 1 fibroma of tendon sheath, 1 giant cell tumor of bone, 1 dedifferentiated liposarcoma, and 1 nonspecific spindle cell tumor. By reconsiderations of our two pathologists, the revised diagnoses were 12 PMTMCTs, 1 giant cell rich PMTMCT, and 1 malignant PMTMCT which was hypercellular and cytologically atypical.

**Figure 2:**
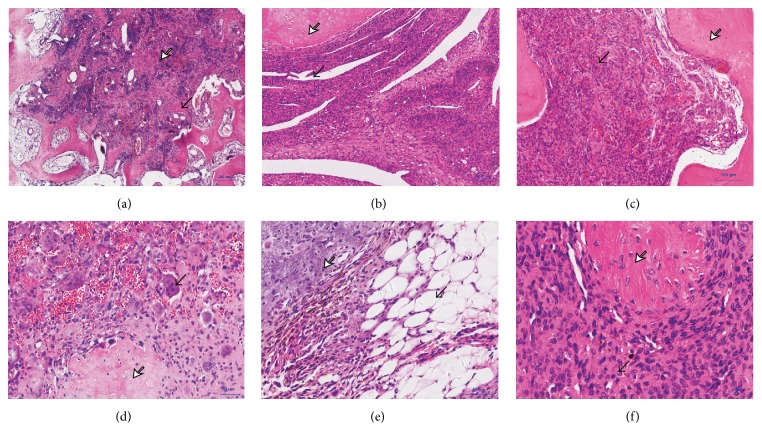
Representative hematoxylin and eosin staining of the PMTMCTs. (a) Infiltrative growth and destructive manner of the tumor. Black arrowhead indicates the destruction of bone trabeculae. White arrowhead indicates that a large number of osteoclast-like giant cells are scattered in the area of bone destruction. (b) Abundant blood vessels with “staghorn” appearance are presented. Black arrowhead indicates the “staghorn” vessels. White arrowhead indicates the cartilage formation. (c) Tumor cells are arranged around the capillaries. Black arrowhead indicates the nested arrangement of tumor cells. White arrowhead indicates the peripheral woven bone shell. (d) Osteoclast-like giant cells are scattered particularly where there is abundant hemorrhage. Black arrowhead indicates the osteoclast-like giant cells. White arrowhead indicates the chondroid matrix. (e) Fat cells, calcifications, and hemosiderin pigmentation could be found in most cases. Black arrowhead indicates the fat cells. White arrowhead indicates the calcifications. (f) The tumor cells and osteoid formation are shown. The tumor cells are usually small, oval-spindle shaped without atypia and embedded in a distinctive matrix. Black arrowhead indicates the oval shaped tumor cells. White arrowhead indicates the osteoid.

The histopathologic feature of the 13/14 benign TIO tumors was similar from case to case wherever the tumor was located. As presented in Figure 2, the tumor cells were oval to spindle shaped, had scarce cytoplasm, and were with fuzzy cell borders. The nuclei were small and vesicular–spindle shaped, with normochromatic and inconspicuous nucleoli. The cytoplasm could be presented with hyalinization changes in some cases, especially the tumors located in bone. Osteoclast-like giant cell was another characteristic of PMTMCTs which was presented in 11/14 cases. Another prominent feature was its abundant blood vessels which was presented in 8/14 cases.

### 3.5. Quantitative ELISA-Like Immunohistochemistry Findings

**Figure 3:**
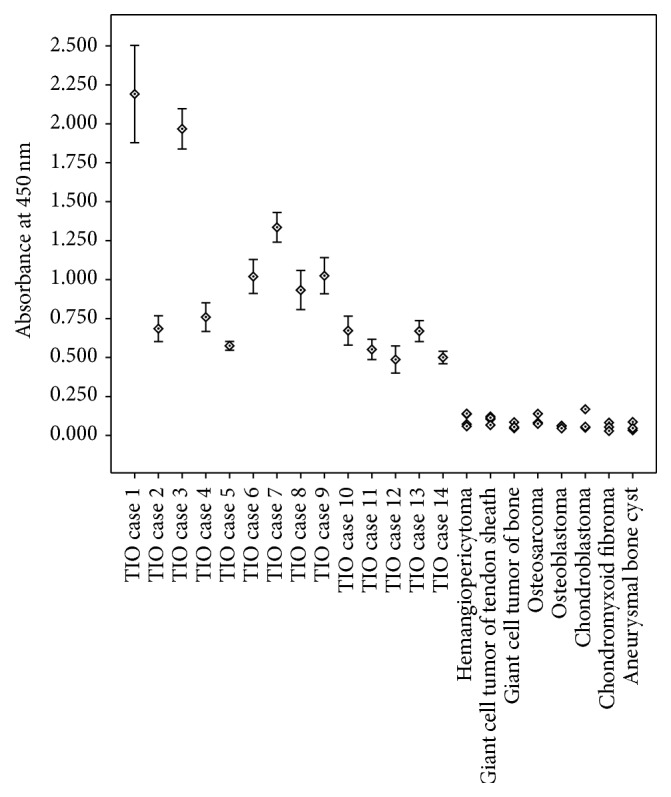
FGF23 levels measured by quantitative ELISA-like immunohistochemistry using paraffin-embedded tissues of 14 TIO cases and 26 non-TIO cases.

Besides the 14 PMTMCTs, 26 mesenchymal tumors where PMTMCTs were usually misdiagnosed were also included, including 4 hemangiopericytomas, 4 giant cell tumors of tendon sheath, 3 giant cell tumors of bone, 3 osteosarcomas, 3 osteoblastomas, 3 chondroblastomas, 3 chondromyxoid fibromas, and 3 aneurysmal bone cysts. As presented in Table 2 and Figure 3, all the TIO cases had high FGF23 expression measured by the quantitative ELISA-like immunohistochemistry (mean 0.99 ± 0.56, range 0.49–2.19, unit: absorbance at 450 nm). Compared with the 26 non-TIO tumors, significant difference could be found (TIO cases of median 0.69, 25%–75% interquartile 0.57–1.10; non-TIO cases of median 0.07, 25%–75% interquartile 0.05–0.11, *p* < 0.001, Mann-Whitney test). The results of non-TIO tumors were as follows: hemangiopericytomas 0.10 ± 0.05, giant cell tumors of tendon sheath 0.10 ± 0.02, giant cell tumors of bone 0.06 ± 0.02, osteosarcomas 0.10 ± 0.04, osteoblastomas 0.06 ± 0.01, chondroblastomas 0.09 ± 0.07, chondromyxoid fibromas 0.05 ± 0.03, and aneurysmal bone cysts 0.05 ± 0.03.

### 3.6. RT-PCR Findings

As presented in Table 2, 6/14 TIO cases were excluded for poor mRNA quality. Of the remaining 8 TIO cases, the 313 bp PCR product that spanned all the 3 exons of FGF23 gene could all be detected. No mutations of the corresponding sequence were sequenced. For the 26 non-TIO tumors, RT-PCR experiments were also conducted to detect the sensitivity of the quantitative ELISA-like immunohistochemistry. However, negative result of FGF23 mRNA transcription was detected in 15/26 tumors while the other 11/26 tumors were excluded from PCR experiment for the poor mRNA quality extracted.

## 4. Discussion

TIO is a rare acquired paraneoplastic disorder characterized by hypophosphatemia and osteomalacia [1–5, 8, 9]. The reported cases here were comprised of 5 cases of initially diagnosed TIO/PMTs and 9 cases with typical clinical and laboratory features of TIO. We reported a novel, feasible, and reproducible FGF23 detecting method of quantitative ELISA-like immunohistochemistry using formalin-fixed and paraffin-embedded tissues. Quantitative ELISA-like immunohistochemistry was previously performed in 96 wells [18, 19], and we simplified the procedure on the slide (but not in 96 wells) and made it more convenient. By performing on 14 TIO tumors and 26 non-TIO tumors, all the TIO tumors had high FGF23 expression and significant difference could be detected (*p* < 0.001). The results were also confirmed by the previous reported RT-PCR assay. As mRNA is usually degraded in paraffin-embedded tissues and RT-PCR experiments usually fail, the quantitative ELISA-like immunohistochemistry for FGF23 expression could be a more practical way for the diagnosis of TIO.

Summarized by the 14 cases, TIO patients were characterized by a long-standing history of osteomalacia (3.9 ± 2.5 years) in most cases (13/14). The initial symptom was usually nonspecific bone pain which would progress gradually. Bone pain of the whole body, walking difficulty, muscle weakness, and osteoporosis would be developed in 2.0–3.0 years. Osteoporotic fractures were common and eventually patients were bedridden. The biochemical manifestations included low serum phosphate level (0.52 ± 0.09 mmol/L), high serum ALP, normal or mild low serum calcium, normal or high intact PTH, high urine phosphate, and low TmP/GFR (0.46 ± 0.10 mmol/L). After complete tumor resection, the renal phosphate wasting and clinical symptoms would be resolved. Bone pain would disappear at 1.0–2.0 months (1.3 ± 0.3 months) and patients usually could walk again at 0.5–3.5 months (1.8 ± 1.9 months). Of the 12/14 patients who underwent intact tumor resection, only 1 patient developed local recurrence during the follow-up period of 0.7–8.0 years (3.0 ± 2.1 years).

While most of the TIO tumors are small slow-growing tumors which frequently situated in unusual sites, identification of the responsible tumor is of challenge and conventional examination usually fails [3, 20]. As mesenchymal cells often express somatostatin receptors type 2 which is of high affinity and specificity to octreotide (a somatostatin analogue), whole-body In-111 octreoscan/pentetreotide scintigraphy has been successfully proposed (positive in 79% of TIO patients [21]). We also identified a promising result in 11/11 cases of octreotide scanning by which 4 tumors were localized. It was also reported that, for the unresectable tumors, octreotide therapy might be useful [22]; however, as in our case  2, poor effect was presented and the same result was also reported in the literature [21].

PMTMCTs are usually misdiagnosed as other tumors, such as vascular tumors (hemangiopericytoma and hemangioma), giant cell tumors, osteoblastoma, chondromyxoid fibroma, chondroblastoma, and osteosarcoma [2–4]. As shown in the present study, only 5/14 cases were initially diagnosed as TIO or PMT, and the other 9 cases were misdiagnosed as other tumors. The idea that most TIO-associated tumors (84.3%) were PMTMCTs was raised by Folpe et al. in 2004 [4]. The proportion of PMTMCTs in the present study was even higher (100%, including 1 giant cell rich PMTMCT and 1 malignant PMTMCT). Since TIO were often delay-diagnosed or misdiagnosed, clinicians and pathologists should be aware of the disease of TIO and PMTMCT, respectively.

FGF23 is an important phosphaturic factor that inhibits inorganic phosphate reabsorption at the renal proximal tubule [14]. As abundant FGF23 protein can be produced by most TIO tumors [2, 11, 12] and the elevated serum FGF23 concentration decreases dramatically after complete tumor removal (the reported plasma half-life was 46–58 min [10]), the quantitative ELISA-like immunohistochemistry reported here is a feasible, reproducible procedure to detect the high FGF23 expression in paraffin-embedded biopsies or surgical specimens. By performing on 14 TIO tumors and 26 non-TIO tumors, all the TIO tumors had high FGF23 expression. By RT-PCR detection for FGF23 transcription in the non-TIO tumors, negative result was detected in 15/15 tumors. As the unit of quantitative ELISA-like immunohistochemistry was absorbance at 450 nm against a reagent blank, the result of 26 non-TIO tumors of a median 0.07, 25%–75% interquartile 0.05–0.11, could be considered as negative FGF23 expression or under detection limit.

The present study reported a large series of TIO cases from a single center. Besides the comprehensive description of clinical, laboratory, and pathological characteristics of the 14 cases, we documented a novel FGF23 detecting procedure. Because the commercial antibody of FGF23 does not work well for conventional immunohistochemistry staining (including the antibody used in the present study) and mRNA is usually degraded for RT-PCR procedure, the technique of quantitative ELISA-like immunohistochemistry could be a useful technique to detect the high FGF23 expression in paraffin-embedded tumor specimens. We simplified the quantitative ELISA-like immunohistochemistry on the slide and made it more convenient. The technique could also be generalized to many other situations in surgical pathology.

The study had several limitations. First, there was no corresponding fresh specimen, no preoperative and postoperative serum available (only available in 1 patient and previously reported elsewhere [17]: the preoperative serum FGF23 level was 417.2 ± 52.7 RU/mL while the corresponding FGF23 expression measured by quantitative ELISA-like immunohistochemistry was 0.55 ± 0.07). Second, as there were only 14 TIO cases and 26 non-TIO cases included and no standard FGF23 detecting technique available in paraffin-embedded tissues, the sensitivity and specificity of the procedure were difficult to calculate. Third, result of quantitative ELISA-like immunohistochemistry was affected both by FGF23 expression level of each tumor cell and by density of the tumor cells. Moreover, although we had identified that the spectrophotometric absorbance of HRP substrate was in direct proportion to the amount of reacted HRP substrate (data not shown), the ratio of quantitative ELISA-like immunohistochemistry to FGF23 expression in the sample was still under further research.

## 5. Conclusion

Quantitative ELISA-like immunohistochemistry was a feasible and reproducible procedure to detect and quantify the high FGF23 expression in formalin-fixed and paraffin-embedded biopsies or specimens and it could provide new insights into the diagnosis and differential diagnosis of TIO associated tumors. Since TIO was often delay-diagnosed or misdiagnosed, clinicians and pathologists should be aware of TIO and PMTMCT, respectively.

## Figures and Tables

**Table 1:** Clinical findings of the included 14 TIO cases.

Cases	Age and gender	Osteomalacia duration (year)	Osteoporotic fracture	Tumor location	Tumor size (cm)	Tumor detection technique	Octreotide scanning	Original pathological diagnosis	Revised pathological diagnosis	Followup
Case 1	19 M	2.5	None	Femoral head	3*∗*2.5	Magnetic resonance imaging	Positive	Nonspecific spindle cell tumor	PMTMCT	Recurrence due to surgery residue and total hip arthroplasty performed at 1.5 m. No further recurrence and normal chemistry at 2.1 y
Case 2	43 F	4	Vertebral compression fracture (3 cm shorter in height); bilateral metacarpus and bilateral pubis fracture	Temporal bone and sphenoid bone	1*∗*1	Octreotide scanning	Positive	PMTMCT	PMTMCT	Unresectable recurrence at 3 m. With carotid artery and cavernous sinus inflicted, no further surgery could be performed. No effect of octreotide therapy. Followup 1.5 y
Case 3	32 F	3.5	Multiple rib fractures	Soft tissue between the second and third metatarsal bone	3*∗*3	Palpation	NA	Giant cell tumor of tendon sheath	PMTMCT	No recurrence and normal chemistry at 7.2 y
Case 4	32 M	10	Vertebral compression fracture (7 cm shorter in height); bilateral femoral heads necrosis; femoral neck fracture	Fifth metatarsal bone	14*∗*9	Ultrasound	Positive	Fibroma of tendon sheath	PMTMCT	Local recurrence at 2.5 y, reoperation performed and normal chemistry at 3.5 y
Case 5	36 M	1.5	Multiple rib fractures	Lateral thigh	4*∗*3.7	Palpation	Positive	PMT	PMTMCT	No recurrence and normal chemistry at 1.8 y
Case 6	40 F	6	None	Subcutaneous tissue of the low back	1.7*∗*1	Palpation	NA	Hemangioendothelioma	PMTMCT	No recurrence and normal chemistry at 8.0 y
Case 7	43 F	4.5	Vertebral compression fracture (15 cm shorter in height); rib fracture	Proximal humerus	4*∗*4	Normal radiography	Positive	Dedifferentiated liposarcoma	Malignant PMTMCT	No recurrence and normal chemistry at 2.1 y
Case 8	42 F	2	Bilateral femoral heads necrosis	Nasal cavity and ethmoid sinus	2*∗*2	Octreotide scanning	Positive	PMT	PMTMCT	No recurrence and normal chemistry at 1.5 y
Case 9	55 F	1.5	None	Gastrocnemius	2*∗*1.5	Palpation	Positive	PMT	PMTMCT	No recurrence and normal chemistry at 0.7 y
Case 10	44 M	3	Distal fibula fracture	Heel	3*∗*2	Palpation	Positive	Giant cell tumor of tendon sheath	PMTMCT	No recurrence and normal chemistry at 2.8y
Case 11	45 F	3.5	Vertebral compression fracture; bilateral femoral heads necrosis; bilateral femoral neck fractures; multiple rib fractures	First metacarpal bone	3*∗*3	Palpation	Positive	Giant cell tumor of tendon sheath	PMTMCT	No recurrence and normal chemistry at 2.1 y
Case 12	45 M	2	Vertebral compression fracture (4 cm shorter in height)	Fibular head	2*∗*1	Octreotide scanning	Positive	Angiofibroma	PMTMCT	No recurrence and normal chemistry at 3.5 y
Case 13	18 F	None	None	Pubic branch and iliac bone	2*∗*1.5	Computerized tomography	NA	Giant cell tumor of bone	Giant cell rich PMTMCT	No recurrence and normal chemistry at 2.0 y
Case 14	52 M	7	None	Chest wall	3*∗*3	Octreotide scanning	Positive	PMT	PMTMCT	No recurrence and normal chemistry at 2.8 y

M: male; F: female; NA: not available; PMT: phosphaturic mesenchymal tumor; PMTMCT: phosphaturic mesenchymal tumor mixed connective tissue variant.

**Table 2:** Laboratory findings of the included 14 TIO cases.

Patient	Serum phosphate (mmol/L, reference 0.89–1.60)	Serum ALP (U/L, reference 0–130)	Serum calcium (mmol/L, reference 2.25–2.75)	Serum intact PTH (pmol/L, reference 1.60–6.90)	Urine phosphate (mmol/24 h)	Urine calcium (mmol/L, reference 2.5–7.5)	TmP/GFR (mmol/L, reference 0.80 to 1.35)	^*∗*^FGF23 expression (absorbance at 450 nm)	^*∗∗*^FGF23 RT-PCR
Case 1	0.34 ± 0.03	687.4 ± 54.0	2.35 ± 0.02	25.07	13.2	2.52	0.29	2.19 ± 0.31	Positive
Case 2	0.53 ± 0.18	231.1 ± 26.9	2.17 ± 0.05	7.55	15.5	1.17	0.45	0.68 ± 0.08	Poor mRNA quality
Case 3	0.51	65.4 ± 1.3	2.18 ± 0.11	6.70	30.5	1.60	0.36	1.97 ± 0.13	Poor mRNA quality
Case 4	0.38 ± 0.06	199.7 ± 4.4	2.30 ± 0.13	4.32	8.5	1.36	0.31	0.76 ± 0.09	Positive
Case 5	0.67 ± 0.03	471.0 ± 19.5	2.32	4.39	27.3	1.19	0.57	0.58 ± 0.03	Poor mRNA quality
Case 6	0.60 ± 0.09	162.8 ± 34.0	2.28 ± 0.09	7.48	21.6	1.20	0.56	1.02 ± 0.11	Positive
Case 7	0.61 ± 0.08	374.9 ± 21.5	2.11 ± 0.06	3.62	5.4	0.60	0.58	1.34 ± 0.1	Positive
Case 8	0.59 ± 0.10	323.2 ± 50.0	2.31 ± 0.09	5.38	26.4	7.28	0.58	0.93 ± 0.13	Positive
Case 9	0.45 ± 0.03	351 ± 12.7	2.14 ± 0.08	7.10	35.0	1.17	0.31	0.68 ± 0.12	Positive
Case 10	0.52 ± 0.10	333.2 ± 35.2	2.26 ± 0.03	9.42	15.6	2.48	0.45	0.67 ± 0.09	Positive
Case 11	0.55 ± 0.19	179.7 ± 14.8	2.15 ± 0.13	4.41	19.1	1.85	0.47	0.55 ± 0.07	Poor mRNA quality
Case 12	0.51 ± 0.05	141.4 ± 25.5	2.30 ± 0.11	3.99	16.0	1.18	0.44	0.49 ± 0.09	Poor mRNA quality
Case 13	0.50	64.1	2.37	NA	13.4	NA	0.45	0.67 ± 0.07	Positive
Case 14	0.49 ± 0.11	131.9	2.20 ± 0.18	14.36	20.2	1.00	0.42	0.50 ± 0.04	Poor mRNA quality

ALP: alkaline phosphatase; PTH: parathyroid hormone; TmP/GFR: maximum tubular resorption of phosphorus factored for glomerular filtration rate; FGF23: Fibroblast Growth Factor 23.

^*∗*^FGF23 was measured by the quantitative ELISA-like immunohistochemistry from formalin-fixed and paraffin-embedded tissues.

^*∗∗*^FGF23 PT-PCR was measured using the formalin-fixed and paraffin-embedded tissues.
